# Doxorubicin-Loaded Delta Inulin Conjugates for Controlled and Targeted Drug Delivery: Development, Characterization, and In Vitro Evaluation

**DOI:** 10.3390/pharmaceutics11110581

**Published:** 2019-11-06

**Authors:** Lixin Wang, Yunmei Song, Ankit Parikh, Paul Joyce, Rosa Chung, Liang Liu, Franklin Afinjuomo, John D. Hayball, Nikolai Petrovsky, Thomas G. Barclay, Sanjay Garg

**Affiliations:** 1 Centre for Pharmaceutical Innovation and Development, School of Pharmacy and Medical Sciences, University of South Australia, Adelaide SA 5000, Australia; lixin.wang@mymail.unisa.edu.au (L.W.); May.Song@unisa.edu.au (Y.S.); Ankit.Parikh@unisa.edu.au (A.P.); Rosa.Chung@unisa.edu.au (R.C.); olumide.afinjuomo@mymail.unisa.edu.au (F.A.); Tom.Barclay@unisa.edu.au (T.G.B.); 2 Division of Biological Physics, Chalmers University of Technology, SE-412 96 Gothenburg, Sweden; Paul.Joyce@unisa.edu.au; 3 Experimental Therapeutics Laboratory, University of South Australia Cancer Research Institute, Adelaide SA 5000, Australia; Liang.Liu@unisa.edu.au (L.L.); john.hayball@unisa.edu.au (J.D.H.); 4 Robinson Research Institute and Adelaide Medical School, University of Adelaide, Adelaide SA 5005, Australia; 5 Vaxine Pty Ltd., Bedford Park, Adelaide 5042, Australia; nikolai.petrovsky@flinders.edu.au; 6 Department of Diabetes and Endocrinology, Flinders University, Adelaide 5042, Australia

**Keywords:** inulin, advax, doxorubicin, anticancer therapy, targeted delivery, pH sensitive, intracellular drug release

## Abstract

Delta inulin, also known as microparticulate inulin (MPI), was modified by covalently attaching doxorubicin to its nanostructured surface for use as a targeted drug delivery vehicle. MPI is readily endocytosed by monocytes, macrophages, and dendritic cells and in this study, we sought to utilize this property to develop a system to target anti-cancer drugs to lymphoid organs. We investigated, therefore, whether MPI could be used as a vehicle to deliver doxorubicin selectively, thereby reducing the toxicity of this antibiotic anthracycline drug. Doxorubicin was covalently attached to the surface of MPI using an acid–labile linkage to enable pH-controlled release. The MPI-doxorubicin conjugate was characterized using FTIR and SEM, confirming covalent attachment and indicating doxorubicin coupling had no obvious impact on the physical nanostructure, integrity, and cellular uptake of the MPI particles. To simulate the stability of the MPI-doxorubicin in vivo, it was stored in artificial lysosomal fluid (ALF, pH 4.5). Although the MPI-doxorubicin particles were still visible after 165 days in ALF, 53% of glycosidic bonds in the inulin particles were hydrolyzed within 12 days in ALF, reflected by the release of free glucose into solution. By contrast, the fructosidic bonds were much more stable. Drug release studies of the MPI-doxorubicin in vitro, demonstrated a successful pH-dependent controlled release effect. Confocal laser scanning microscopy studies and flow cytometric analysis confirmed that when incubated with live cells, MPI-doxorubicin was efficiently internalized by immune cells. An assay of cell metabolic activity demonstrated that the MPI carrier alone had no toxic effects on RAW 264.7 murine monocyte/macrophage-like cells, but exhibited anti-cancer effects against HCT116 human colon cancer cells. MPI-doxorubicin had a greater anti-cancer cell effect than free doxorubicin, particularly when at lower concentrations, suggesting a drug-sparing effect. This study establishes that MPI can be successfully modified with doxorubicin for chemotherapeutic drug delivery.

## 1. Introduction

Targeted drug release has received considerable interest to achieve the controlled release of therapeutic drug doses at target sites, thereby reducing peak systemic concentrations and the risks of dose-related side effects and toxicity [1,2]. Several bioconjugates have displayed promising targeted release characteristics by coupling drug molecules with specific carrier systems, including liposomal systems [3,4], proteins [5,6], polymers [7], and nanoparticles [8,9]. Each conjugate offers specific advantages and limitations, and no single method emerges as a universal platform [10]. However, non-toxic polymeric carriers possess several benefits over other systems, including high stability, both in vivo and during storage, versatility, and control over chemical and physical properties. Of these, natural polysaccharides have been identified as a promising alternative to synthetic polymeric systems, as their high biocompatibility and biodegradability overcomes limitations of some synthetic polymers [11]. Furthermore, natural polysaccharides are often significantly cheaper to source and may be easier to manipulate than other carriers [12]. Their many hydroxyl groups and other chemistries can also act as a scaffold for chemical modification and drug attachment [13,14].

Inulin is a natural polysaccharide comprised of linear chains of fructose groups capped at the reducing end with glucose [15,16]. MPI, a specific semicrystalline particulate form known as delta inulin or Advax™, can specifically bind to and be internalized by monocytes, macrophages, and dendritic cells with high efficiency [17]. The immunomodulatory properties of delta inulin have been exploited to develop a potent vaccine adjuvant [18,19,20,21,22,23,24], and has also shown benefit in anticancer treatment [25,26]. A previous study showed that doxorubicin conjugated to soluble inulin using a carboxymethylation method enabled the cytotoxic response to be maintained or improved at lower doses as compared to free doxorubicin [27]. The key advantages of using inulin particles in MPI rather than free soluble inulin, which was reported by Peppas and colleagues in 2013 [27], could further help drug conjugate effectiveness by preventing rapid renal excretion, increasing the biological half-life, and enabling uptake of the injected MPI particles by monocytes with subsequent selective tissue transport to lymphoid organs [28,29]. Consequently, we hypothesized that MPI might be a promising targeted drug delivery vehicle for immune cell cancers, such as myeloid leukemias.

Doxorubicin is used in chemotherapy regimens for many cancers. Due to its risk of cardiotoxicity, clinicians often need to lower the dose, which may decrease its effectiveness. We sought to design an acid–labile covalent linkage system to attach doxorubicin to the nanostructured MPI surface. In this system, a covalent bond is formed, ameliorating the risk of a burst release mechanism, which is common for monocyte-targeted liposomal systems [30]. Once transported into monocytes, the drug-modified MPI particles should be hydrolyzed by acidic conditions in the lysosomes [31]. Released doxorubicin may then remain within the monocytes or exit through diffusion or via cell membrane carriers and enter the surrounding tissue [32]. The MPI-doxorubicin conjugate was characterized by FTIR and SEM and the in vitro release profiles at varying pH were investigated using the dialysis method. The in vitro cellular uptake and anticancer activity of the MPI-doxorubicin conjugate was also performed. The reported results show the ability to use MPI-doxorubicin particles as a promising drug delivery system.

## 2. Materials and Methods

### 2.1. Materials

The MPI was from Vaxine Pty Ltd. (Adelaide, Australia) and was produced as described previously [19], and doxorubicin was from Melonepharma (Dalian, China). Saline (sodium chloride for injection BP 0.9%) was from InterPharma (Sydney, Australia). Daunorubicin, methanol, ammonium dihydrogen phosphate, acetic acid, zinc sulfate, acetonitrile *N*-(3-dimethylaminopropyl)-*N*′-ethylcarbodiimide hydrochloride (EDC), *N*-hydroxysuccinimide (NHS), Fetal Bovine Serum (FBS), penicillin G, streptomycin, MTT (3-(4,5-Dimethylthiazol-2-yl)-2,5-Diphenyltetrazolium Bromide), and all materials required to prepare Artificial Lysosomal Fluid (ALF) and Simulated Body Fluid (SBF) were from Sigma-Aldrich. Phosphate buffered saline (PBS, pH 7.4, 0.01 M) solutions were prepared from Sigma-Aldrich tablets and all solutions were prepared using pure water (18.2 MΩ). Succinic anhydride and dimethyl sulfoxide (DMSO) were from Merck (Bayswater, Australia). DMEM culture media, McCoy′s Modified Media, l-glutamine, formalin, 4′,6-diamidino-2-phenylindole (DAPI), fluorescent mounting media, and trypsin were from ThermoFisher Scientific Australia. All chemicals were of analytic grade and used as received.

### 2.2. Methods

#### 2.2.1. Synthesis of Doxorubicin-Loaded Microparticulate Inulin (MPI)

##### Modification of MPI Particles with Succinic Anhydride

Succinic anhydride (0.400 g, 4.0 mM) was added to a gently stirred suspension of MPI (0.26 g, ~4.92 mM–OH groups) and DMAP (40 mg, 65.5 mM) in pyridine (5.0 mL, 26.8 mM) at 0 °C. The temperature was maintained at 0 °C for 6 h before being increased to room temperature and the reaction continued for a further 42 h. The mixture was then diluted with PBS (6 mL) and centrifuged (rcf = 3000, 90 s) before discarding the supernatant. The solid was then washed twice with methanol (rcf = 3000, 30 s) and three times with PBS buffer (rcf = 4500, 90 s) to remove all traces of pyridine and other reagents. Parameters that were varied to optimize succinate functionalization of MPI were: Pyridine concentration, succinic anhydride concentration, and reaction times.

##### Doxorubicin Attachment to MPI-Succinate Particles

Succinate-modified MPI (50 mg, ~0.95 mM–OH groups) was dispersed in sodium phosphate buffer (pH 7.4, 0.1 M, 5 mL). Doxorubicin was added to this dispersion in 5-fold molar excess (21 mg, 0.039 mM), along with EDC in 10-fold molar excess (15.6 mg, 0.078 mM) and NHS to make 5 mM solution (2.9 mg, 0.025 mM). The reaction was continued for 3 h at room temperature. The unbound doxorubicin was removed by centrifuging (rcf = 1000, 30 s). The MPI was washed by centrifuging (rcf = 4500, 90 s) and replacing the supernatant with PBS until all unbound doxorubicin was removed. Optimization of doxorubicin coupling was achieved by varying initial doxorubicin and EDC concentrations.

#### 2.2.2. Physicochemical Characterization of Doxorubicin-Loaded MPI

##### FTIR

Attachment of doxorubicin to MPI was confirmed with Fourier transform infrared spectroscopy (FTIR) using a Thermo Electron Corporation Nicolet 6700 spectrophotometer (Medison, WI, USA) with a DGTS TEC detector using 64 scans at a resolution of 4 cm^−1^. Data manipulation was conducted using OMNIC^TM^ series software (Thermo Fisher Scientific, Waltham, MA, USA).

##### NMR

Coupling of Doxorubicin molecules to MPI was confirmed using Proton Nuclear Magnetic Resonance (^1^H NMR) spectroscopy. Approximately 20 mg of DOX-modified MPI was washed twice in D2O before the pellet was dissolved in DMS series O-d6. ^1^H NMR spectra were obtained using an UltraShieldTM 300 (Bruker, Billerica, MA, USA). The acquisition parameters were as follows: Spectral width 6410 Hz, relaxation delay 3 s, number of scans 64, acquisition time 4.819 s, and pulse width 90°.

##### SEM

SEM was used to confirm that the synthesis did not significantly alter the size or morphology of the modified MPI compared to unmodified particles. The particle size and surface morphology of unmodified and modified MPI were examined by high-resolution analytical scanning electron microscopy (SEM) (Zeiss Merlin, Oberkochen, Germany). Each sample was mounted on double-sided adhesive tape and sputter coated with a gold layer (~5–10 nm) prior to imaging at an accelerating voltage of 1–2 kV.

#### 2.2.3. Determination of Doxorubicin Loading

Drug loading was investigated using HPLC. MPI-doxorubicin (1 mg) was weighed and suspended in HCl (1 mL, 0.1 M) and incubated at 37 °C with continuous shaking for 6 h. The sample was then centrifuged (10,000 rcf for 10 min) and the supernatant was taken and diluted 10-fold by the HPLC mobile phase mixture before injection into the HPLC system. The concentration of doxorubicin was analyzed by HPLC (Shimadzu Corporation, Kyoto, Japan) using a C18 column (250 × 460 mm) and a PDA detector (start λ 200 nm, end λ 350 nm, analyzed at 250 nm) [33]. The mobile phase was methanol/ammonium dihydrogen phosphate 0.01 M (NH_4_H_2_PO_4_)/acetic acid/pure water (70:17:0.5:12.5%). The flow rate of the mobile phase was 1.0 mL/min and the injection volume was 20 μL. The linearity of the method was checked linearity in water samples from 0.01–1 μg/mL and 1–30 μg/mL, resulting in a high observed linearity with *R*^2^ > 0.999. All measurements were conducted in triplicate and the mean values and standard deviations are reported.

#### 2.2.4. In Vitro Cleavage of MPI

##### Preparation of Artificial Lysosomal Fluid (ALF) and Simulated Body Fluid (SBF)

For the preparation of ALF and SBF, the materials in Table S1 (Supporting Information) were added to 1 L pure water and mixed until completely dissolved [34]. ALF solutions with pH values of 4.5, 5.2, and 6.0 were prepared by adjusting the sodium hydroxide concentration in the mixture (Figure S1).

##### Cleavage of MPI

MPI was dispersed in ALF (15 mL, pH 4.5) at a concentration of 10 mg/mL. The medium was kept at 37 °C and stirred continuously at 200 rpm. Aliquots (500 μL) were periodically taken and were centrifuged immediately. The supernatant was neutralized by addition of saturated sodium bicarbonate solution (60 μL) to inhibit further acid-mediated hydrolysis. Both the supernatant and pellet were retained for analysis. The concentration of fructose, glucose, and sucrose cleaved from MPI was analyzed using a HPLC system (Shimadzu Corporation, Kyoto, Japan) consisting of a series of LC-20ADXR pumps, SIL-20ACXR auto sampler, CTO-20AC column oven set at 30 °C, ELSD-LTII evaporative light scattering detector, and a Luna amino analytical column (NH2, 5 μm, 4.6 mm ID × 250 mm). The mobile phase was a mixture of acetonitrile and pure water (95:5 *v*/*v*), eluted at a flow rate of 1.0 mL/min. The limit of detection (LOD) of the analytical method was 20 μg/mL for all sugars. Linear calibration curves (*R*^2^ ≥ 0.99) were plotted for chromatographic peak areas against sugar concentrations over the range of 33–1000 μg/mL, without the addition of an internal standard. All analytes were diluted suitably to meet the calibration concentration range.

#### 2.2.5. Study of Doxorubicin Release from MPI

MPI-doxorubicin (200 mg) particles were dispersed in pure water (10 mL). The suspension was vortexed and a sample (300 μL) was added to a dialysis bag (MWCO 1.4 k Da) and left to dialyze in ALF or SBF release medium (15 mL). The dialysis system was kept at 37 °C and stirred continuously at 20 rpm using a Benchtop 808C Incubator Orbital Shaker (Adelab Scientific, Adelaide, SA 5067, Australia). Release medium (1 mL) was collected from the outside of the dialysis bag at scheduled intervals and replaced with an equal amount of fresh release medium. The removed samples were kept at −20 °C and protected from light until analysis. The release of MPI-doxorubicin was compared to a pure doxorubicin control in which doxorubicin (10 mg) was dissolved in pure water (10 mL). The solution (100 μL) was then transferred to a dialysis bag and the dialysis and sample collection were the same as for the MPI-doxorubicin. As the doxorubicin was released from MPI-doxorubicin, free doxorubicin was evaluated by HPLC using aliquots (200 μL) from the removed samples and following the validated method described below. All measurements were conducted in triplicate and the mean value and standard deviations are reported.

##### Validation of the Analytical Method

The method was validated following the International Conference on Harmonisation (ICH) of Technical Requirements for Registration of Pharmaceuticals for Human Use, Validation of Analytical Procedures: Text and Methodology Q2 (RI). The method was validated for specificity, linearity, accuracy, precision, limit of detection (LOD), and limit of quantification (LOQ) levels. The linearity of the standard curve for doxorubicin was evaluated by preparing calibration standards (*n* = 3) each day for 3 days. The precision of the method was assessed by analyzing the intra-day and inter-day variability on the same day (*n* = 6) and on three different days at a concentration of 8 μg/mL. Accuracy was calculated by comparing the concentration determined at 10, 20, and 30 μg/mL (*n* = 3) of samples. The limit of quantification (LOQ) was determined experimentally from the lowest concentration which had a signal-to-noise ratio superior to 10, and the limit of detection (LOD) was determined from the lowest concentration that has a signal-to-noise ration greater than 3 times.

##### Sample Preparation and Data Analysis

A stock solution of doxorubicin was prepared in pure water (1.0 mg/mL). The stock solution was then diluted with pure water to prepare working solutions at a variety of final concentrations. The calibration standards of doxorubicin were prepared by spiking the appropriate amount of standard solution in pure water. All standard samples were stored at −20 °C and protected from light until analysis.

Analysis software used was version LC solution, LabSolutions (Shimadzu, Tokyo, Japan). Data analysis was carried out using Microsoft Excel and Graph Pad Prism version 7.02.

#### 2.2.6. Cell Culture Conditions

For in vitro studies, the Raw 264.7 murine macrophage line (RAW cells), RAW-Blue reporter cells, and the human colorectal cancer cell line, HCT116 were used. Briefly, cells were maintained at 37 °C, 5% CO_2_ in DMEM and McCoy’s Modified Media, respectively. All media used contained 10% Fetal Bovine Serum (FBS), penicillin G (100 units/mL), streptomycin (100 μg/mL), and 10 mM l-glutamine.

#### 2.2.7. Cellular Uptake of Doxorubicin

The cellular uptake behavior and the intracellular distribution of the free doxorubicin and MPI-doxorubicin were analyzed by both confocal laser scanning microscopy (CLSM) and flow cytometry (FACS).

##### Cell Internalization Observations

RAW 264.7 cells were counted and 1 × 10^5^ cells/well were plated into each 12-well plate. All plates were then left overnight for attachment in 37 °C, 5% CO_2_. Cells were then exposed to free doxorubicin, MPI-doxorubicin treatments at 50 μg/mL for 30, 120, and 240 min. At the end of each time point, cells were washed 3 times in sterile PBS and then scraped off and spun down at 1000 rpm for 5 min. Cell pellets were then resuspended in 100 μL of FBS. Resuspended cells were then spun down onto labeled superfrost glass slides using a cytocentrifuge. Briefly, cells were spun down at 1000 rpm for 5 min using Cytospin4 cytocentrifuge (Thermo Fisher, Waltham, MA, USA) to ensure an even and uniform preparation of cells before staining. Slides were then left to dry for 24 h before fixation for 10 min in neutral buffered formalin. Counterstaining and coverslipping were then performed using DAPI containing fluorescent mounting media. Images of each time point and treatment were captured by using a Zeiss Elyra laser scanning confocal microscope (Carl Zeiss, Jena, Germany) with Zeiss ZEN lite software.

##### Flow Cytometry Measurements

For flow cytometry, RAW 264.7 and HCT 116 cells were seeded in 12-well plates at a density of 1 × 10^5^ cells per well and cultured in high glucose and modified McCoy′s 5A media containing 5% FBS, l-glutamine, penicillin G, and streptomycin respectively for 24 h in 37 °C, 5% CO_2_. The free doxorubicin and MPI-doxorubicin were then dissolved in RAW and HCT 116 culture medium with the doxorubicin concentration of 50 μg/mL and were added to different wells. The cells were incubated at 37 °C for 30, 120, and 240 min, and the samples were then prepared for flow cytometry analysis. More specifically, wells containing HCT 116 cells were rinsed with PBS three times before trypsinization. Following trypsinization, cells were collected and centrifuged at 1000 rpm for 5 min. The supernatant was removed, and the pellets were resuspended with 200 μL of PBS. Similarly, treated RAW cells were rinsed 3 times in PBS, scraped off and spun down for 5 min at 1000 rpm. The supernatant was also removed, and pellets resuspended in 200 μL PBS for flow cytometry analysis. Data for 20,000 gated events were collected and analyzed by a Beckman Coulter CytoFlex flow cytometer (Beckman Coulter, CA, USA) with the PE fluorescence. The results were analyzed with FlowJo software (Becton, Dickinson and Company (BD), Warwick, RI, USA) and the intensity is shown on a four-decade log scale.

#### 2.2.8. In Vitro Cytotoxicity Assay

First, 5 × 10^3^ cells were plated in 100 μL of media in a 96-well plate and left overnight. Following 24 h of incubation at 37 °C, 5% CO_2_, medium was removed and replaced with doxorubicin or doxorubicin–MPI in varying concentrations of 20, 10, 5, 1, 0.1, 0.01 μg/mL. In addition to doxorubicin and doxorubicin–MPI conjugate, serial concentrations of MPI alone were also done (1, 0.5, 0.25, and 0.125 mg/mL) to observe any cytotoxic effects of the carrier particles. Cells were then left at 37 °C, 5% CO_2_ to incubate for a further 24, 48, or 72 h. To check the viability of cells, MTT assays were then performed. Media was removed and replaced with 100 μL of fresh media and 50 μL of MTT. Following 4 h of incubation, MTT was removed and DMSO was added. Plates were then read at an absorbance of 450 nm on a Perkin Elmer Wallac plate reader (PerkinElmer Inc, Waltham, MA, USA) [35,36].

## 3. Results/Discussion

### 3.1. Synthesis and Characterization of MPI-Doxorubicin

**Figure 1:**
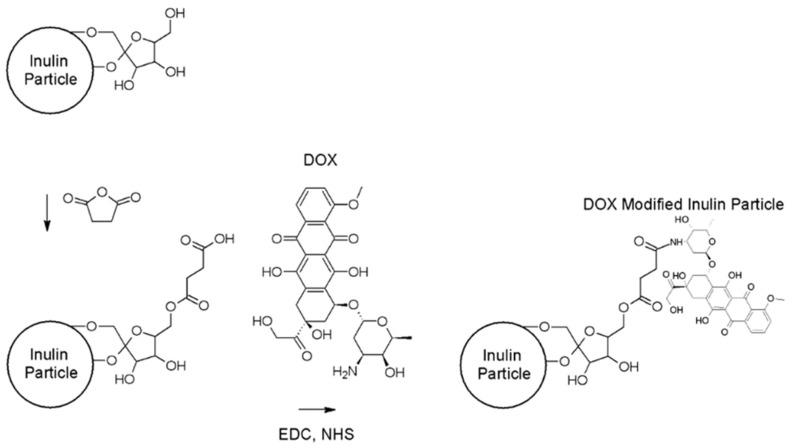
Doxorubicin-modified microparticulate inulin (MPI).

Doxorubicin was successfully coupled to MPI through a two-step process. MPI was firstly modified with succinic anhydride to initiate the synthesis of doxorubicin-modified MPI. Succinic anhydride reacts with hydroxyl groups on the MPI, creating an ester linkage between the succinate group and the MPI. The carboxylic acid of succinate-modified MPI particles was then activated with 1-Ethyl-3-(-3-dimethylaminopropyl) carbodiimide to form an active *O*-acylisourea intermediate. This intermediate was then displaced by a nucleophilic attack from the primary amine group of doxorubicin, resulting in doxorubicin-modified MPI (Figure 1) and a soluble urea derivative by-product [37]. The succinic derivative of INU has been widely described before in the literature as well [38,39,40].

#### 3.1.1. Analysis of MPI-Doxorubicin Using FTIR and NMR

**Figure 2:**
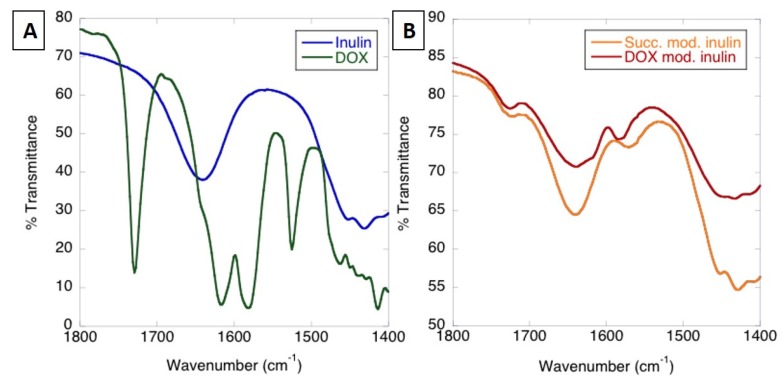

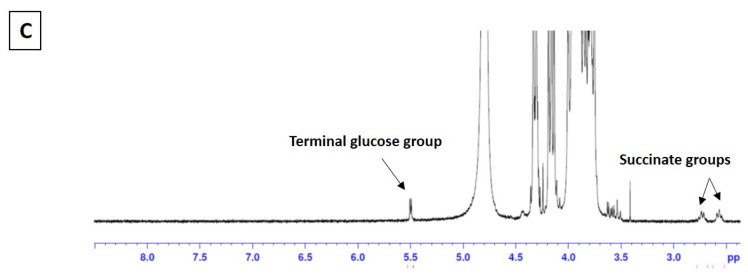
Characterization of MPI-Doxorubicin (**A**) FTIR MPI and doxorubicin; (**B**) FTIR succinate-modified inulin and MPI-doxorubicin conjugate, (**C**) ^1^H NMR spectra of succinic anhydride-functionalized MPI particles.

The attachment of doxorubicin to MPI was characterized using Fourier transform infrared spectroscopy (FTIR). MPI modified with anhydrides, including succinic anhydride, has been shown to provide additional peaks in between 1500 and 1700 cm^−1^ [41], attributed to asymmetric stretching of carbonyl groups of carboxylate (1571 acid) and ester groups (1724 ester) [42,43]. These peaks appear in succinic anhydride-modified MPI as shoulders to the O–H deformation peak of incorporated water at 1641 cm^−1^ (Figure 2A,B) [44]. Previous analysis of succinyl doxorubicin showed that the doxorubicin peaks are generally much lower absorbing than the carbonyl peaks of the succinyl group, explaining the minimal difference with the doxorubicin attachment [45]. Nonetheless, there are small changes in the character of the MPI O–H peak and –CH_2_ scissoring and C–O–H deformation peaks in the region of 1440 cm^−1^ that can be attributed to the presence of doxorubicin (Figure 2A). There was also a shift in the peak for succinate carboxylate group at 1571 to 1585 cm^−1^, attributable to amide (II) transitions and demonstrating the successful attachment of the doxorubicin to MPI succinate through an amide bond [46]. The ^1^H NMR also showed a peak at ~4.4 ppm and no observable additional peaks at ~5.35–5.65 ppm, indicating that the inulin particles were preferentially substituted at the C6 position of fructose [47].

##### Optimization of Doxorubicin Attachment to MPI

**Figure 3:**
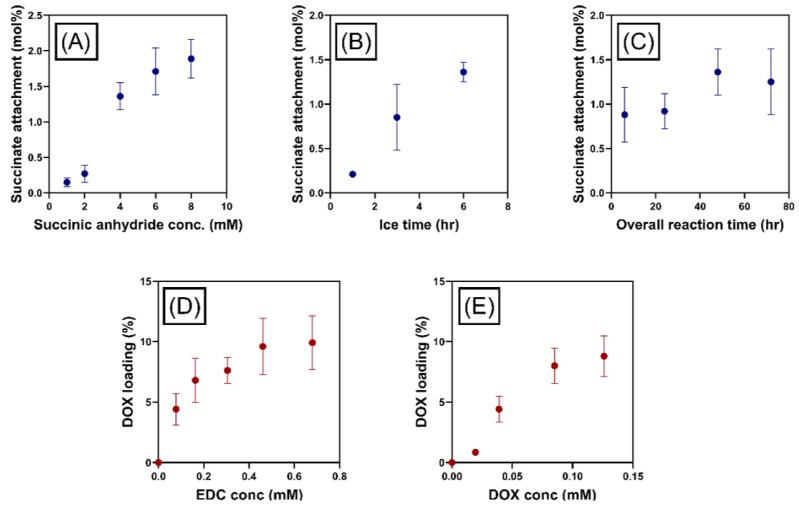
The number of succinate groups attached (mol%) as a function of: (**A**) Succinic anhydride concentration, (**B**) reaction time at 0 °C, and (**C**) overall reaction time. The reaction times used were: 6 h at 0 °C, 48 h reaction time. The doxorubicin loading (%) as a function of (**D**) EDC concentration and (**E**) doxorubicin concentration.

The degree of succinate attachment to MPI was enhanced by varying the concentration of succinic anhydride initially added to the solution, succinate attachment increasing linearly as a function of initial succinic anhydride concentration (Figure 3A). The concentration of succinate groups was measured in relative terms to the number of inulin chains, which was determined by comparisons of the number of anomeric glucose groups (1 per inulin chain) and succinate groups observed in ^1^H NMR spectra (Figure 2C). The degree for the succinic anhydride attachment was conducted by comparing the peak at around 2.7 with the inulin peak between *δ* = 3.5 and 4.25 [48] The ^1^H of –CH_2_CH_2_ in the succinyl group has a chemical shift around 2.43–2.70 ppm. This approach has been used to quantify the degree of succinic anhydride in previous study [47]. Pitarresi et al. synthesized inulin-based macromolecular derivatives, which were exploited for iron deficiency anemia treatment. The intermediate product was inulin succinate. Vermeersch et al. reported the synthesis of inulin monosuccinate where the same way of characterization was used.

Other variables tested for the succinate attachment to MPI included those relating to reaction time. The yield of succinate groups was increased by increasing the time of reaction on ice (Figure 3B) for given overall reaction time. The influence of overall reaction time on succinate loading was less pronounced compared to other reaction variables. However, the attachment was still enhanced with increasing reaction time, until a maximum was reached at 48 h (Figure 3C). The volume of pyridine used in this reaction was also varied to investigate reaction volume on succinate attachment, but this was found to have no effect. Consequently, the ideal reaction conditions used in this study were considered to be: 8.0 mM succinate reacted for 6 h at 0 °C and 48 h overall reaction time. Doxorubicin attachment to MPI-succinate particles increased with increasing initial EDC and doxorubicin concentrations (Figure 3D,E). In both cases, the concentration of doxorubicin loaded to MPI plateaued to a maximum drug loading determined by UV spectroscopy, which was likely controlled by the number of succinate groups attached to the MPI. The maximum drug load was achieved using doxorubicin and EDC concentrations of 25 mM and 69 mM, respectively.

The conjugation chemistry can result in the formation of chemical linked MPI-doxorubicin, as well as a small proportion of supramolecular absorbed doxorubicin on the surface of the nanostructured MPI. It should be noted that the characterization techniques utilized in this study (i.e., the combination of ^1^H NMR and FTIR) are limited with regard to determining the ratio of chemically linked doxorubicin versus supramolecularly bound doxorubicin that was present within the MPI. Subsequently, this was accounted for when analyzing the release behavior of doxorubicin from MPI. Ultimately, it is not an issue if the doxorubicin is simply adsorbed into the INU, as long as it does not result in burst release prior to reaching the target site.

In this study, EDC chemistry was selected over hydrazone chemistry due to the ability to covalently attach a significantly higher amount of succinate groups to the MPI, when compared with hydrazone groups. As a result, doxorubicin loading was significantly greater when EDC chemistry was used. Moreover, hydrazone chemistry is much less stable, particularly at neutral pH. This is why we did not attach doxorubicin using that method.

#### 3.1.2. Size and Morphology by SEM

The morphology of MPI (Figure S2A,B) and MPI-doxorubicin (Figure S2C,D) was examined using SEM. Particle size was consistent between MPI and MPI-doxorubicin at approximately 1.5 μm diameter. SEM images (Figure S2) demonstrated that the current synthesis protocol for MPI-doxorubicin had no visible impact on the physical nanostructure and integrity of the MPI physical structure, suggesting that this might be a suitable coupling methodology that maintains the immune properties of the MPI.

### 3.2. In Vitro Hydrolysis of MPI

MPI used in this experiment was delta inulin isoform [49] with an average chain length of 39 fructose units capped at the reducing end with a glucose group, as calculated by end group analysis using ^1^H NMR spectroscopy [50]. Since the glycosidic bonds of MPI are acid–labile [50], the stability of the MPI was tested under the pH conditions expected in mature lysosomes, matching the most acidic conditions used in drug cleavage studies. This built upon previous work conducted over 21 days [28]; in this case, the rate of hydrolysis of MPI in acidic ALF (pH 4.5) was monitored over 165 days by observing the release of MPI component units: Fructose, sucrose, and glucose (Figure S3).

Fructose release kinetics occurred roughly linearly with time, and the rate of ~15–18 μg/mL per day was maintained throughout the reaction period. The final concentration of fructose in the cleavage media after 165 days was 2.75 mg/mL, which corresponds to the release of 28.3% of all fructose groups in the MPI. Glucose release kinetics were equivalent to fructose over the first 7 days under the cleavage conditions, despite the much lower amount of glucose (2.6%) in MPI compared to fructose (97.4%), and at 7 days, 41% of all glucose groups and only 1.2% of fructose groups had been cleaved from the MPI. By 39 days, in ALF, ~95% of all glucose groups had been cleaved from the MPI.

Previously, it has been hypothesized that MPI formation is initiated from a bidentate glucose to fructose intermolecular hydrogen bonding interaction [51], followed by the organization into antiparallel helices of inulin chains aligned orthogonally within the two-dimensional crystalline nanolayers that make up the three-dimensional MPI [52]. This theory places the glucose groups at the surface of the layered structure and explains the initial rapid release of glucose compared to fructose. Consequently, these experimental results further justify our previous model of MPI formation and the importance of glucose to particle formation.

The overall hydrolysis of MPI is plotted in Figure S3. This demonstrates that after 165 days, approximately 30% of the original MPI was hydrolyzed. At this point, the dispersion was still a cloudy suspension, indicative of the presence of remaining intact MPI despite the loss of essentially all of the glucose from the delta inulin particles. Further, SEM of the remaining inulin particles after 21 days of incubation showed that they remained intact and looked essentially unchanged [28]. Hence, although it was previously demonstrated that the glucose end group of inulin is critical for MPI formation [52], these new results show that the subsequent loss of the glucose from the inulin polymer does not unduly affect MPI structure. Nonetheless, the relatively rapid removal of fructose and glucose groups from the surface of the MPI particles under acidic conditions might be relevant to the release of covalently attached drugs under the same conditions, as the drugs will also be attached to the surface glucose and fructose groups.

### 3.3. Doxorubicin Loading and Release Evaluations

#### 3.3.1. Analytical Method Development and Validation

The chromatographic conditions to analyze doxorubicin were optimized to achieve good resolution and symmetric peak shapes of analytes, as well as minimizing the run time. This included extensive experimentation with the composition of the mobile phase. Figure S4 shows the chromatographic profiles of a standard sample of doxorubicin in water. Under the optimized conditions, the best and fastest separation was achieved by mobile phase containing 70% methanol. With this composition of mobile phase, doxorubicin was eluted at 5.6 min, the peak at 2.8 min was the solvent peak. A mobile phase of the same composition was also suitable for doxorubicin analysis of tissue samples. This method was further developed for the quantitative analysis of doxorubicin in plasma, blood, urine, and tissue samples.

The HPLC method was checked using doxorubicin dissolved in pure water samples from 0.01–1 μg/mL and 1–10 μg/mL, providing a high observed linearity with *R*^2^ > 0.999 (Table S2). Intra- and inter-day variability (Table S3). The RSD of the between 0.06–1.25%, with the recovery of 97.56–100.15% (Table S4). All the accessed parameters were within the acceptable limits according to ICH guidelines. Results demonstrate that the developed method to quantify doxorubicin is reproducible, accurate and reliable. The LOQ was found to be 1.56 ng/mL. The limit of detection (LOD) was determined to be 0.3 ng/mL. The LOD and LOQ levels were comparable or superior to those methods in the literature that used fluorescence detection.

#### 3.3.2. Percentage of Drug Loading Determination

For the optimized MPI-doxorubicin particles, the drug loading was measured using HPLC. It was shown that 2.48 ± 0.12% *w*/*w* of doxorubicin was loaded onto the surface of the MPI.

#### 3.3.3. Release Profiles of Doxorubicin-Loaded MPI in the Different Release Medium

ALF was used as biorelevant media for the in vitro release of MPI-doxorubicin particles, mimicking the conditions inside maturing lysosomal compartments of monocytes. The pH of release media was varied to determine the influence of pH on the rate and extent of doxorubicin release, and SBF was used as a control release media, simulating neutral biological conditions. The release was measured in triplicate at a constant temperature of 37 °C to approximately human body temperature.

**Figure 4:**
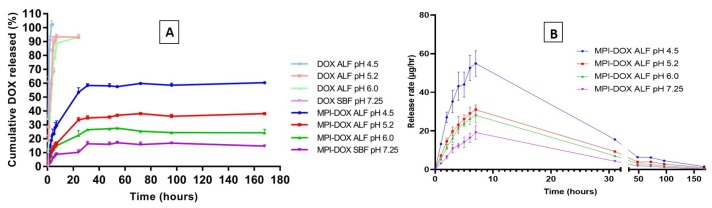
(**A**) Cumulative doxorubicin release profiles from free doxorubicin and the pH-dependent doxorubicin release from MPI-doxorubicin by dialysis in ALF and SBF at 37 °C. (**B**) Release profile of doxorubicin from MPI-doxorubicin in different pH release medium. Data are presented as the mean ± SD (*n* = 3).

It was found that as acidity in the release medium increased, the extent and rate of doxorubicin release from MPI-doxorubicin formulation increased significantly. The in vitro total cumulative percentage released of doxorubicin from the MPI-doxorubicin conjugate is shown in Figure 4A. Approximately 60% of doxorubicin was released from MPI-doxorubicin after 168 h in ALF at pH 4.5, 38% was released in pH 5.2, and 24% of doxorubicin was released in pH 6.0. Compared to release in ALF, the doxorubicin release was significantly reduced in pH 7.25 SBF and only 11% of doxorubicin was released from MPI-doxorubicin formulation by 168 h. The transport and detection from the dialysis tube to the external media within 7 h in acidic ALF conditions. In the neutral conditions of SBF, the transport of doxorubicin was also rapid, but only 67% of doxorubicin was released, most likely due to deprotonation of the amine group reducing doxorubicin solubility. Comparison of the control and MPI-doxorubicin results show that the release rate of doxorubicin from MPI-doxorubicin conjugate was controlled, with a significant dependence of release rate and total release on pH, confirming the effectiveness of the controlled drug release system.

Figure 4B plots release rate against time and shows an initial burst release in the first 7 h, with more doxorubicin released at a faster rate at lower pH values. The pH of the ALF solutions was chosen to replicate conditions of endosomes (pH 6.0) developing into lysosomes (pH 4.5), in which there are also enzymes that can cleave the covalent linkages. In the first 3 h, 9% of doxorubicin was released from the MPI-doxorubicin in SBF pH 7.25. This was most likely caused by the immediate dissolution of supramolecularly bound doxorubicin rather than doxorubicin covalently bound to the outer surface of MPI. The initial burst release is a very common problem in most delivery systems, which can cause toxicity, shorter half-life, and therefore require more frequent administration. With the MPI-doxorubicin delivery system, the particles are likely to stay in the SBF conditions for an extended period of time and so only suffer from ~9% burst release that occurs in SBF. Slow uptake into macrophages and the time taken for lysosomes to form will gradually subject the particles to the lower pH and enzymes that cleave the linkage between the MPI and doxorubicin. The release rate was slower after 10 h, but still significant up to approximately 48 h, after which release was very slow up to the final measured point at 168 h.

MPI-doxorubicin demonstrated pH-modulated release below the pKa of doxorubicin, confirming that the release behavior was not triggered by electrostatic interactions, but by cleavage of the covalent linkages between doxorubicin–succinite and MPI. The succinyl moiety can still be linked to the primary amine of doxorubicin, since cleavage is hypothesized to occur at the acid–labile ester bond. While a limitation of this study was the inability to assess the cleavage of the succinyl moiety from the parental doxorubicin molecule, due to the absence of endosomal enzyme, previous studies have demonstrated the rapid cleavage kinetics of amide bonds by endosomal enzymes [53]. Subsequently, the authors predict that the succinyl moiety will be completely cleaved from doxorubicin within the endosome.

Overall, the doxorubicin release profiles from MPI-doxorubicin in the buffers followed the acidic pH-dependent release and up to 6 times more doxorubicin (Figure 4B) was released from MPI-doxorubicin in monocyte lysosomal conditions (to 60% released in ALF pH 4.5 versus 11% released from SBF). This demonstrates selective release and that the proposed transport and release of doxorubicin from doxorubicin–MPI is realistic. In this way, doxorubicin–MPI could raise the therapeutic index and the convenience of doxorubicin administration in cancer chemotherapy.

### 3.4. Cellular Uptake of MPI-Doxorubicin by Monocytes Cells

**Figure 5:**
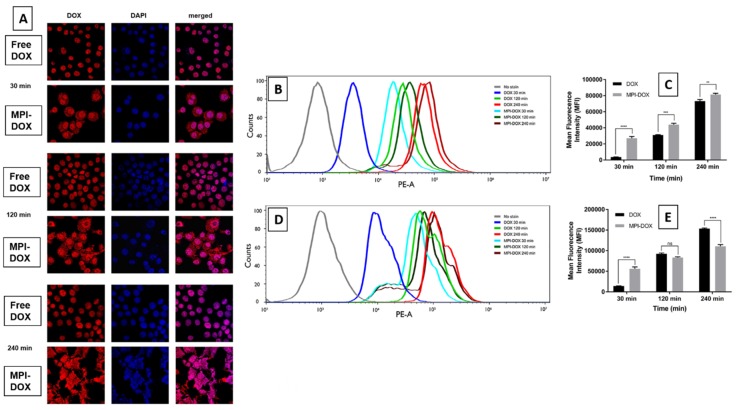
(**A**) CLSM image of RAW 264.7 cells incubated with free doxorubicin and MPI-doxorubicin for various times. For each panel, the images from left to right show fluorescence of doxorubicin in cells (red), cell nuclei stained by DAPI (blue), and overlays of two images. Flow cytometry analysis of doxorubicin fluorescence intensity incubated with free doxorubicin and MPI-doxorubicin at an equivalent concentration of doxorubicin at 50 μg/mL under 37 °C for 30, 120, and 240 min. The bar length represents 10 μm. (**B**) histogram profile of RAW cells; (**C**) mean fluorescence intensity (MFI) in RAW cells; (**D**) histogram profile of HCT 116 colon cancer cells; (**E**) mean fluorescence intensity (MFI) in HCT 116 cells. The data are expressed as the mean ± SD (*n* = 3), ** *P* < 0.01, *** *P* < 0.001 and **** *P* < 0.0001 (two-way or one-way ANOVA, Tukey’s test).

To confirm the cellular uptake of MPI-doxorubicin and the intracellular release of doxorubicin, the naturally fluorescent property of doxorubicin was used for confocal laser scanning microscopy of uptake into RAW cells. For free doxorubicin, a significant amount of fluorescence was observed in the nuclei only, whereas doxorubicin fluorescence was clearly observed in both the cytoplasm and the nuclei for MPI-doxorubicin after 30, 120, and 240 min incubation, with the cells incubated with MPI-doxorubicin swelling to 3–4 times their normal size due to uptake of the particles (Figure 5A). When free doxorubicin was added to the cells, they exhibited no change in size with doxorubicin content by fluorescence approximately equivalent at all time points (Figure 5A). This is because free doxorubicin enters and can exit the cell by a passive diffusion mechanism. In contrast, after incubation with MPI-doxorubicin intense, doxorubicin fluorescence appeared in both the nuclei and cytoplasm of RAW cells (Figure 5A). The obvious fluorescence signal from the cytoplasm indicates that MPI-doxorubicin particles enter the cells via a different mechanism compared to free doxorubicin. In our model, MPI-doxorubicin is slowly taken up by endocytosis and then the doxorubicin released in the endosomal acidic environment from where it migrates to the cytoplasm and then the nucleus. A large number of cells lysed within 240 min of incubation at the concentration of 50 μg/mL, and this is confirmed on the confocal images of these groups showing large dysmorphic dead cells, confirming potent cytotoxicity due to the targeted cellular delivery. The lower frequency of the remaining cells is clearly evident at all time points for the MPI-doxorubicin groups.

The cellular uptake of free doxorubicin and MPI-doxorubicin were quantified by flow cytometry, for both RAW and HCT116 cells. Figure 5B–D shows the histograms of RAW and HCT116 cell doxorubicin fluorescence intensity after incubation with free doxorubicin and MPI-doxorubicin at an equivalent concentration of 50 μg/mL at time intervals of 30, 120, and 240 min. After 30 min incubation, there was an evident enhancement of fluorescence intensity in both RAW and HCT116 cells compared to control cells, indicating the cellular uptake of free doxorubicin and MPI-doxorubicin. However, at 30 min incubation, the mean fluorescence intensity (MFI) of MPI-doxorubicin-treated cells was 5–10 times stronger in both cell lines when compared to cells treated with free doxorubicin. At 120 and 240 min, the significant enhancement of doxorubicin uptake with MPI-doxorubicin was still observed in RAW cells compared to cells treated with free doxorubicin, but with the longer incubation times, the differences in MFI of RAW cells became less. This suggests an enhanced early uptake of MPI-doxorubicin, which then reaches a steady state. This may be based on the number of MPI-doxorubicin particles a cell is able to phagocytose per unit time. By contrast, the uptake of free doxorubicin follows more first-order kinetics based on the drug concentration. In analysis of HCT116 cell cultures, MPI-doxorubicin showed a pronounced toxicity with increased incubation time and at the 240 min time point, there were few viable HCT116 cells remaining (<15% viability) for flow cytometry analysis. Hence MPI-doxorubicin induced greater cytotoxicity than free doxorubicin in HCT116 cells.

MPI-doxorubicin generally gave higher fluorescence intensity after 30 min incubation in the RAW cells. It is known that free doxorubicin can be transported into cells by diffusion, while the MPI-doxorubicin is usually taken up by cells, especially monocytes, via endocytosis, as shown in the CLSM and our previous studies [28]. Doxorubicin, a potent anticancer drug, exerts its effects via intercalation with DNA and inhibition of macromolecule biosynthesis [54,55]. Consequently, it is important that the MPI-doxorubicin is able to be delivered into the cell and then the doxorubicin released from MPI particles into the cytoplasm from whence it can enter the cell nucleus to have its effects.

### 3.5. In Vitro Cytotoxicity

**Figure 6:**
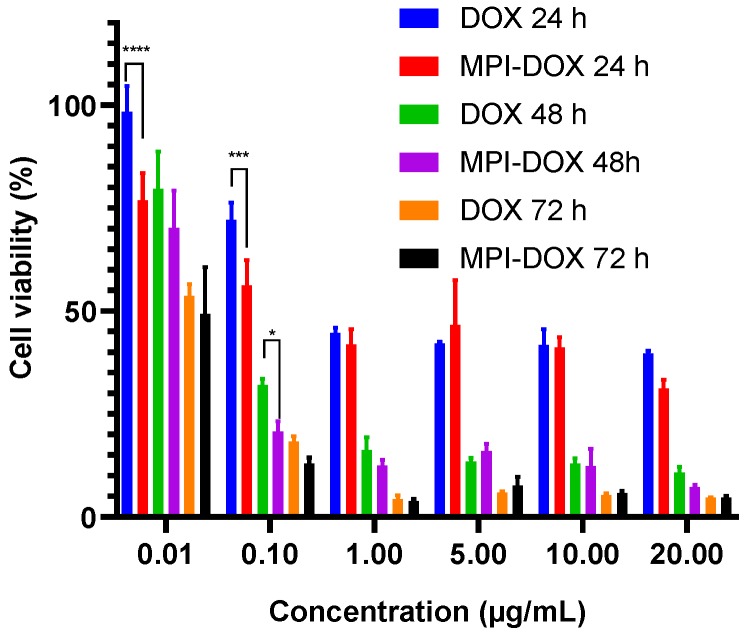
Cell viability % (MTT assay) of free doxorubicin and MPI-doxorubicin at doxorubicin or equivalent concentrations of 0.01, 0.1, 1, 5, 10, 20 mg/mL; free doxorubicin and MPI-doxorubicin on HCT 116 human colon cancer cells with 24, 48, and 72 h incubation time. The results are reported as mean ± SD (*n* = 4), * *P* < 0.05, *** *P* < 0.001 and **** *P* < 0.0001 (two-way or one-way ANOVA, Tukey’s test).

The cytotoxicity of the blank MPI was investigated both in RAW blue cells and in HCT116 cells by MTT assays. The cells were incubated with blank MPI for 24 h and the results revealed that MPI was non-toxic to RAW blue cells at all concentrations tested, with a viability rate higher than 90% at 1 mg/mL (Figure S5). The cytotoxicity of doxorubicin and MPI-doxorubicin is shown in Figure S6 on RAW cell line. MPI-doxorubicin showed low toxicity compared to doxorubicin with monocytes and other phagocytic cells. In HCT 116 cells, MPI at the highest 1 mg/mL concentration showed an unexpected 30% cytopathic effect. The mechanism for this is currently unknown. MPI-doxorubicin at the lowest concentrations of 0.01 and 0.1 mg/mL doxorubicin equivalents showed significantly higher cytotoxicity than free doxorubicin, consistent with a dose-sparing effect, using the different delivery system (Figure 6 and Figure S6). The MTT results were in accordance with the CLSM and flow cytometry studies where doxorubicin from MPI-doxorubicin entered the HCT116 cells more effectively, particularly at lower concentrations, and induced a better cytotoxicity effect than free doxorubicin. This preferential cytotoxicity activity of MPI-doxorubicin versus free doxorubicin on human colon cancer cells was unexpected, as it was initially hypothesized that the MPI-doxorubicin particles would only be phagocytosed by monocytes. Its uptake by and apparently selective cytotoxic action on human colon cancer cells, both when used by itself and to an even greater extent when conjugated with doxorubicin, suggests the existence of a novel pathway by which colon cancer cells might be able to be selectively killed—which warrants further investigation, including testing in other cancer cell line types. Moreover, in vivo work in murine cancer models is warranted to better understand the response to MPI-DOX, particularly if it is injected directly into tumor mass where we could expect both increased uptake and a depot effect unlikely to be seen with free DOX.

## 4. Conclusions

In the present study, doxorubicin was successfully shown, for the first time, to be able to be conjugated to MPI particles using a succinate attachment intermediate. The MPI-doxorubicin particles provided pH-dependent sustained release of doxorubicin. The MPI-doxorubicin particles were rapidly internalized by two different cell lines, the murine RAW monocytic cell line and human HCT116 colon cancer cell line. MPI-doxorubicin had more potent cytotoxic action when compared to free doxorubicin, particularly at early time points and at lower concentrations, consistent with a change in drug delivery kinetics and behavior. This suggests that MPI-doxorubicin particles may have utility for the treatment of tumors, including through, as yet, uncharacterized mechanisms mediated by the inulin particles themselves—In addition to their drug delivery function.

## Supplementary Materials

The following are available online at https://www.mdpi.com/1999-4923/11/11/581/s1, Figure S1: Sodium hydroxide titration in ALF. Figure S2: (A–B) SEM MPI; (C–D) SEM MPI-doxorubicin conjugate. Figure S3. Concentration of (blue ●) fructose, (orange ■) glucose and (green ▲) sucrose as a function of time during MPI hydrolysis in acidic ALF (pH 4.5) over an extended cleavage period. Figure S4. Chromatographic determination of doxorubicin, using HPLC (Shimadzu Corporation, C18 column (250 × 460 mm) and a PDA detector. Figure S5. Cytotoxicity of MPI at carrying concentrations of 0.125, 0.25, 0.5, 1 mg/mL. RAW and HCT 116 tumour cells were incubated with MPI for 24 h. the cell viability was determined by MTT assay (*n* = 3). Figure S6. Cell viability % (MTT assay) of free doxorubicin and MPI-doxorubicin at doxorubicin or equivalent concentrations of 0.01,0.1,1,5,10,20 mg/mL; free doxorubicin and MPI-doxorubicin on RAW Blue cells with 24, 48 and 72 h incubation time; Table S1. Reagents for preparing ALF (pH 4.5, 5.2 and 6.0) and SBF. Table S2. Linearity of the developed assay method. Data are presented as the mean ± SD (*n* = 3). Table S3 Intra-day and Inter-day precision of the developed assay method. Table S4 Analytical recovery of MPI by developed method.
