# Supplementary material for: Doxorubicin-Loaded Delta Inulin Conjugates for Controlled and Targeted Drug Delivery: Development, Characterization, and In Vitro Evaluation
Source: Pharmaceutics. 2019 Nov 6;11(11):581. doi: 10.3390/pharmaceutics11110581 (PMC6920814; doi:10.3390/pharmaceutics11110581)
Supplement: Supplementary file 1 [file pharmaceutics-11-00581-s001.pdf]

pharmaceutics

MDPI

Supplementary Materials: Doxorubicin-Loaded Delta Inulin Conjugates for Controlled and Targeted Drug Delivery: Development, Characterization, and In Vitro Evaluation

Lixin Wang, Yunmei Song, Ankit Parikh, Paul Joyce, Rosa Chung, Liang Liu, Franklin Afinjuomo, John D. Hayball, Nikolai Petrovsky and Thomas G. Barclay and Sanjay Garg

Table S1. Reagents for preparing ALF (pH 4.5, 5.2 and 6.0) and SBF.

Material	ALF	SBF
	**Concentration (g/L)**	**Concentration (g/L)**
Sodium hydroxide	Varying with pH	-
Citric acid	20.8	-
Hydrochloric acid (1 M)	-	7.8 mL
Sodium chloride	3.21	8.04
Sodium bicarbonate	-	0.355
Calcium chloride.2H_2O	0.128	0.292
Potassium chloride	-	0.225
Potassium phosphate dibasic trihydrate	-	0.311
Sodium phosphate $Na_2HPO_4.2H_2O$	0.089	-
Sodium sulfate	0.039	0.072
Magnesium chloride.6H_2O	0.106	0.311
Glycerol	0.059	-
Sodium citrate dihydrate	0.077	-
Sodium tartrate dihydrate	0.090	-
Sodium lactate	0.085	-
Sodium pyruvate	0.086	-
Tris(hydroxymethyl) aminomethane	-	6.12

Table S2. Linearity of the developed assay method. Data are presented as the mean ± SD (n = 3).

Linearity			
Equation	Y = 37498*X − 35344	Y = 34806*X − 32133	Y = 34280*X − 29717
Slope ± SD	37498 ± 312.8	34806 ± 182.3	34280 ± 188.7
Y-intercept ± SD	−35344 ± 4883	−32133 ± 2846	−29717 ± 2947
R square	0.9987	0.9995	0.9994

Table S3. Intra-day and Inter-day precision of the developed assay method.

Concentration added (μg/mL)	Intra-day			Inter-day		
	**Mean peak area**	**% RSD**	**Mean RSD (%)**	**Mean peak area**	**% RSD**	**Mean RSD (%)**
8 (n = 6)	249584.33	0.83		255885.33	1.07	
8 (n = 6)	246023.00	0.75	0.72	246014.11	0.72	0.86
8 (n = 6)	242435.00	0.58		241928.00	0.77	

Table S4. Analytical recovery of MPI by developed method.

Accuracy			
**Concentration added (μg/mL)**	**Concentration measured (mean ± SD)**	**% RSD**	**Recovery (%)**
10 (n = 3)	9.76 ± 0.12	1.25	97.56
20 (n = 3)	20.03 ± 0.02	0.11	100.15
30 (n = 3)	30.02 ± 0.02	0.06	100.08

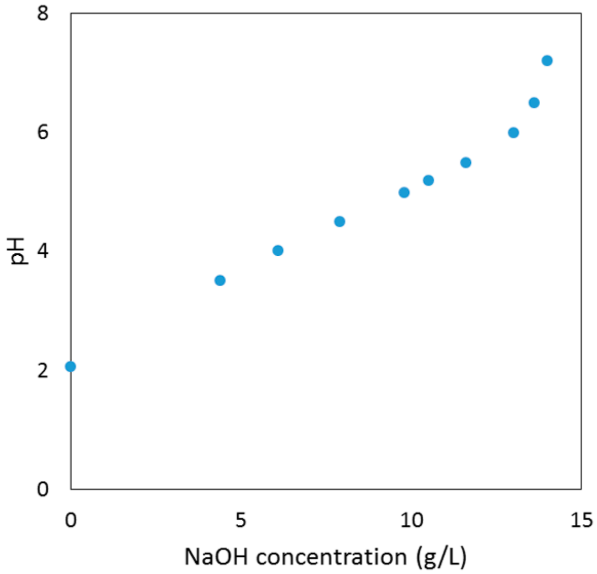

Figure S1. Sodium hydroxide titration in ALF.

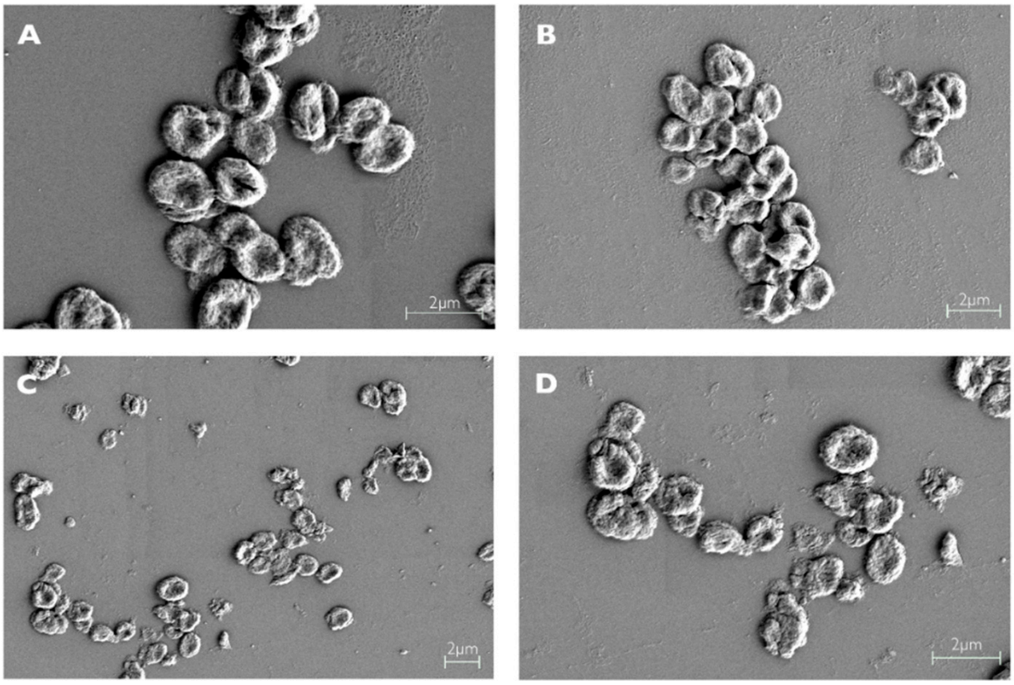

Figure S2. (**A**–**B**) SEM MPI; (**C**–**D**) SEM MPI-doxorubicin conjugate.

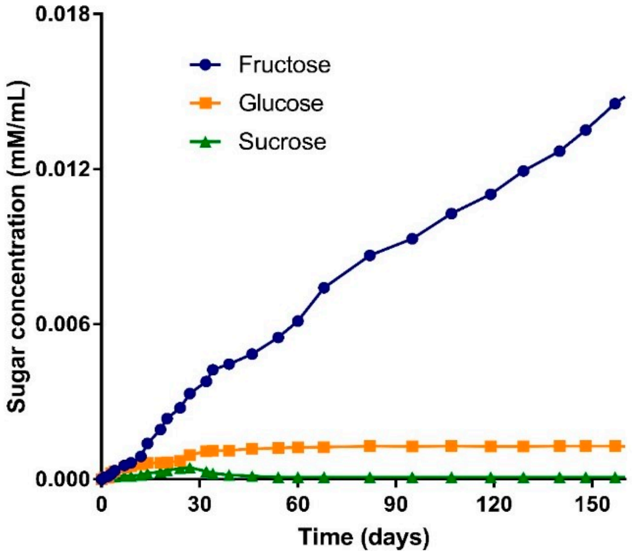

Figure S3. Concentration of (blue ●) fructose, (orange ■) glucose and (green ▲) sucrose as a function of time during MPI hydrolysis in acidic ALF (pH 4.5) over an extended cleavage period.

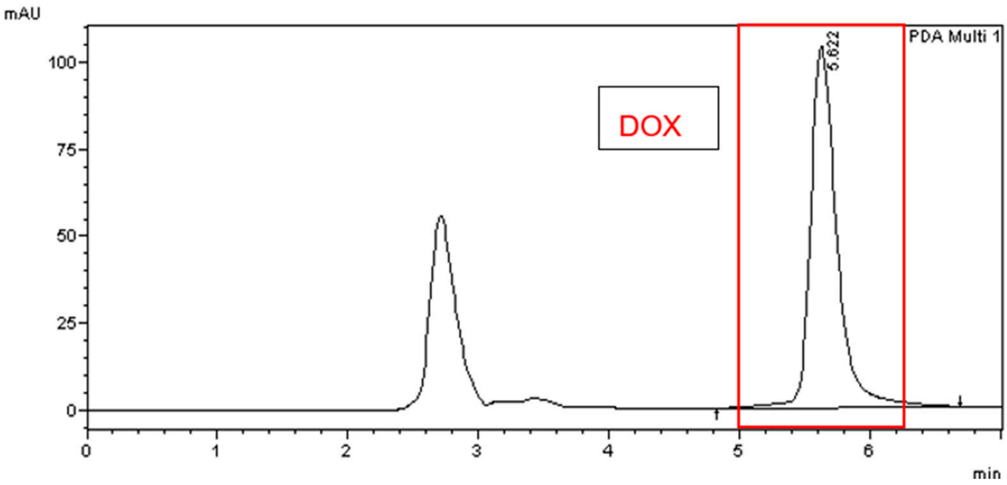

Figure S4. Chromatographic determination of doxorubicin, using HPLC (Shimadzu Corporation, C18 column (250 × 460 mm) and a PDA detector.

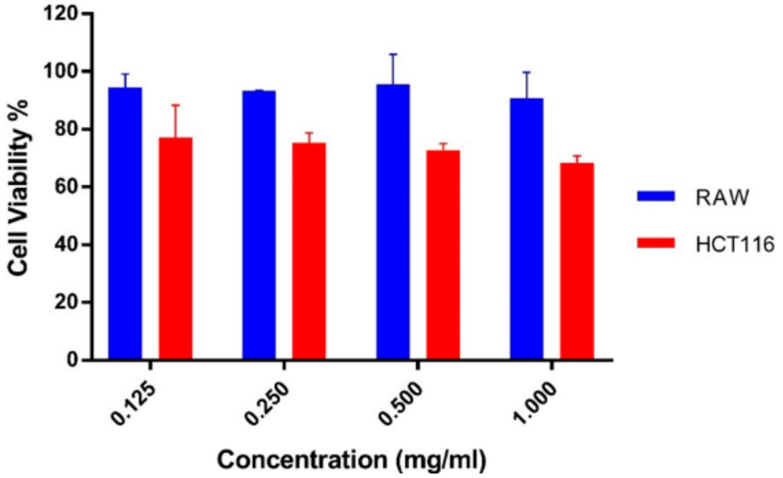

Figure S5. Cytotoxicity of MPI at carrying concentrations of 0.125, 0.25, 0.5, 1 mg/mL. RAW and HCT 116 tumour cells were incubated with MPI for 24 h. the cell viability was determined by MTT assay ($n = 3$).

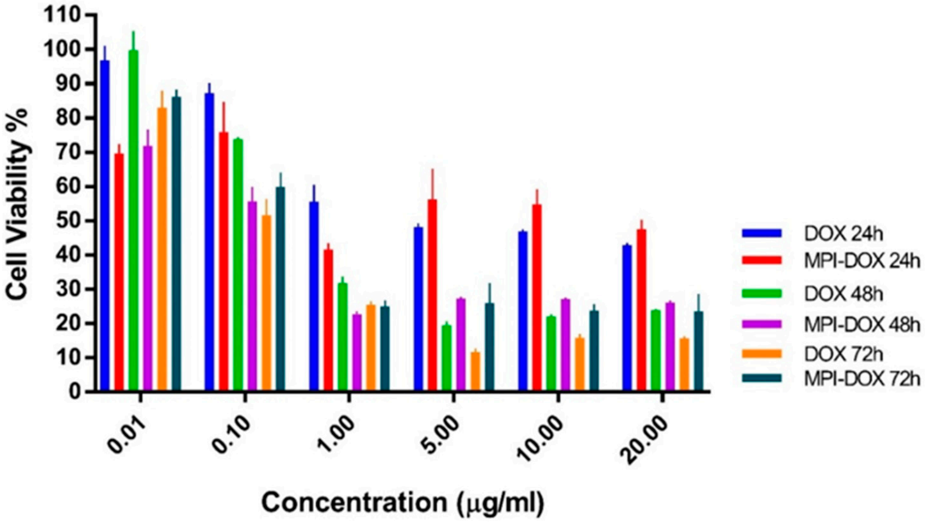

Figure S6. Cell viability % (MTT assay) of free doxorubicin and MPI-doxorubicin at doxorubicin or equivalent concentrations of 0.01,0.1,1,5,10,20 mg/mL; free doxorubicin and MPI-doxorubicin on RAW Blue cells with 24, 48 and 72 h incubation time.
